# Treatment reality in elderly patients with advanced ovarian cancer: a prospective analysis of the OVCAD consortium

**DOI:** 10.1186/1757-2215-6-42

**Published:** 2013-06-28

**Authors:** Fabian Trillsch, Linn Woelber, Christine Eulenburg, Ioana Braicu, Sandrina Lambrechts, Radoslav Chekerov, Els van Nieuwenhuysen, Paul Speiser, Alain Zeimet, Dan Cacsire Castillo-Tong, Nicole Concin, Robert Zeillinger, Ignace Vergote, Sven Mahner, Jalid Sehouli

**Affiliations:** 1 Department of Gynecology and Gynecologic Oncology, University Medical Center Hamburg-Eppendorf, Martinistrasse 52, Hamburg, 20246, Germany; 2 Department of Medical Biometry and Epidemiology, University Medical Center Hamburg-Eppendorf, Hamburg, Germany; 3 Department of Gynecology, European Competence Center for Ovarian Cancer; Campus Virchow Klinikum, Charité – Universitätsmedizin Berlin, Berlin, Germany; 4 Department of Obstetrics and Gynaecology, Division of Gynaecological Oncology, Universitaire Ziekenhuizen Leuven, Katholieke Universiteit Leuven, Leuven, Belgium; 5 Department of Obstetrics and Gynecology, Medical University of Vienna, Vienna, Austria; 6 Department of Obstetrics and Gynecology, Innsbruck Medical University, Innsbruck, Austria

**Keywords:** Ovarian cancer, Prognosis, Elderly, Surgery, Chemotherapy, Performance status

## Abstract

**Background:**

Approximately one third of women diagnosed with ovarian cancer is 70 years or older. Information on the treatment reality of these elderly patients is limited.

**Methods:**

275 patients with primary epithelial ovarian cancer FIGO stage II-IV undergoing cytoreductive surgery and platinum-based chemotherapy were prospectively included in this European multicenter study. Patients <70 and ≥70 years were compared regarding clinicopathological variables and prognosis.

**Results:**

Median age was 58 years (18–85); 47 patients (17.1%) were 70 years or older. The postoperative 60-day-mortality rate was 2.1% for elderly and 0.4% for younger patients (p < 0.001). Elderly patients were less likely to receive optimal therapy (no residual disease after surgery and platinum combination chemotherapy) compared to patients <70 years (40.4% vs. 70.1%, p < 0.001) and their outcome was less favorable regarding median PFS (12 vs. 20 months, p = 0.022) and OS (30 vs. 64 months, p < 0.001). However, in multivariate analysis age itself was not a prognostic factor for PFS while the ECOG performance status had prognostic significance in elderly patients.

**Conclusions:**

Elderly patients with ovarian cancer are often treated less radically. Their outcome is impaired despite no consistent prognostic effect of age itself. Biological age and functional status should be considered before individualized treatment plans are defined.

## Background

Women aged 65 and above represent the fastest-growing population segment with consecutively rising incidence of malignancies [1]. Median age at first diagnosis of ovarian cancer is currently 63 years with approximately one third aged 70 or older [2]. With the progressive demographic changes the percentage of elderly patients will further rise and gynecologic oncologists will have to focus on these patients and their specific needs. Current evidence on the treatment of ovarian cancer in this patient cohort is scarce. Prospective phase III trials concentrating on ovarian cancer therapy usually reveal an underrepresentation of elderly patients as physicians seem to hesitate to enrol these patients even if it is possible according to the study protocol (e.g. AGO OVAR-3 trial with only 13% of patients ≥ 70 years) [3].

Irrespective of age, ovarian cancer is still regarded the most lethal gynecological malignancy with a median overall survival of approximately 44 months [4]. However, even in advanced tumor stage (FIGO [International Federation of Gynecology and Obstetrics] stage ≥ IIB) the intention of treatment is still curative achieving a rate of approximately 20% of patients without relapse after optimal primary treatment [4]. Optimal treatment thereby consists of the combination of radical cytoreductive surgery (resection of all visible tumor) plus platinum-based combination chemotherapy and is associated with significant morbidity [4].

In contrast to the well-established prognostic factors postoperative residual tumor load, FIGO stage at first diagnosis and lymph node involvement the role of age itself for ovarian cancer patients has been controversially discussed in the past. While several studies found higher age to be associated with poor survival [5-7], it could not be proven as an independent prognostic factor in a recent large, retrospective cohort study from Denmark [8]. The reason might be that the impact of age is strongly influenced by other factors such as co-morbidities and suboptimal therapeutic management [8].

To further understand the role of age for outcome and treatment of ovarian cancer we analyzed the cohort of prospectively enrolled patients from the Sixth Framework Programme (FP6) EU project “OVCAD – Diagnosis of a Silent Killer” with focus on age. The project was implemented in 2005 by 5 large European gynecologic cancer centers to identify and verify clinical and molecular prognostic factors for ovarian cancer. The present analysis investigates the prognostic value of age itself as well as possible confounders impairing the prognosis of elderly patients and further elaborates the need for trials specifically designed for age-related questions.

## Methods

### Patients

Patients with advanced epithelial ovarian cancer FIGO II-IV were prospectively enrolled into the OVCAD project between February 2005 and December 2008 by five European Gynecological Cancer Centers (Charité University Medical Center Berlin, Germany; University Hospitals Leuven, Belgium; University Medical Center Hamburg-Eppendorf, Germany; Medical University of Vienna, Austria; and Innsbruck Medical University, Austria). Data from other clinical subprojects of the OVCAD project has recently been published [9-11].

Patients had to be 18 years or older without evidence of any other primary tumor affecting prognosis or treatment of ovarian cancer. Patients were eligible for study entry if radical surgery was performed with the intention of optimal cytoreduction and platinum-based chemotherapy was applied. Systemic treatment could be given either postoperatively or as neoadjuvant treatment. Patients were selected for neoadjuvant therapy if they were not eligible for surgery (e.g. acute thromboembolism) or initial achievement of complete tumor resection was judged to be very unlikely with upfront debulking surgery by the treating gynecologic oncologist in pre-operative evaluation. Complete surgical staging consisted of total hysterectomy and bilateral salpingo-oophorectomy, peritoneal washings, omentectomy, appendectomy, and resection of all visible tumor if possible. All histopathological reports were re-evaluated for the inclusion criteria after surgery to guarantee accurate diagnosis and staging. To measure treatment response, CA-125 levels were assessed before and after treatment completion and the diagnosis of disease recurrence or progression was established based on CA-125 variations (GCIG criteria), imaging studies, and results of performed biopsies [12]. All patients gave written informed consent prior to surgery and collection of any biological samples. The protocol was approved by the local ethical committees of the different centers and by the centralized ethical committee of Vienna (http://www.ovcad.eu). Clinical data were anonymized and collected in a centralized online database (Alcedis GmbH, Germany) solely accessible by the OVCAD partners.

### Statistical analysis

All statistical analyses were conducted using SPSS software version 20.0 (SPSS Inc., Chicago, IL, USA) and Stata 11.0 (2009 StataCorp LP, College Station, Texas, USA). P-values <0.05 were considered statistically significant.

To evaluate the impact of age on treatment and prognosis patients were separated in those <70 years and those ≥70 years as previously proposed [13]. The subgroups were then compared regarding clinicopathological variables and prognosis by applying Man-Whitney-U test, chi square test, Fisher’s exact test or logrank test, as appropriate. Kaplan-Meier method was used to analyze and illustrate survival. For multivariate analysis of the prognostic impact of age and performance status on survival, log-rank tests and Cox regression models were calculated accounting for multicenter analysis. These models were applied separately for the whole patient cohort as well as for elderly patients only.

## Results

A total of 275 patients met the inclusion criteria and were prospectively enrolled into the OVCAD study. Median age of the total cohort was 58 years (range 18–85) with 228 patients younger than 70 years (82.9%, median age 55) and 47 patients aged 70 or older (17.1%, median age 75). The median follow up was 36 months. Detailed clinical patient characteristics are provided in Table 1.

**Table 1:** Clinical patient characteristics

	**Total (%) n = 275**	**Age < 70 years (%) n = 228**	**Age ≥ 70 years (%) n = 47**	**p value for group differences**
**Age (years)**				**<0.001a**
Median	58	55	75	
Range	18-85	18-69	70-85	
**FIGO stage**				0.801c
IIB/C	15 (5.5)	13 (5.7)	2 (4.2)	
IIIA/B	16 (5.8)	13 (5.7)	3 (6.4)	
IIIC	196 (71.3)	165 (72.4)	31 (66.0)	
IV	48 (17.5)	37 (16.2)	11 (23.4)	
**Nodal status**				**0.002b**
pN0	65 (23.6)	58 (24.4)	7 (14.9)	
pN1	143 (52.0)	124 (54.4)	19 (40.4)	
Nx	67 (24.4)	46 (20.2)	21 (44.7)	
**Grading**				0.907c
G1	10 (3.6)	9 (4.0)	1 (2.1)	
G2	64 (23.4)	54 (23.8)	5 (21.3)	
G3	200 (73.0)	164 (72.2)	42 (76.6)	
Unknown	1	0	1	
**Histological subtype**				0.700c
Serous	237 (86.2)	194 (85.1)	43 (91.5)	
Mucinous	3 (1.1)	3 (1.3)	0 (0.0)	
Other*	13 (4.7)	31 (13.6)	4 (8.5)	
**Presence of ascites**				0.964b
None	66 (24.0)	54 (23.7)	12 (25.5)	
<500 ml	101 (36.7)	84 (36.8)	17 (36.2)	
>500 ml	108 (39.3)	90 (39.5)	18 (38.3)	
**Peritoneal carcinomatosis**				0.513b
Yes	186 (67.4)	156 (68.0)	30 (63.8)	
No	89 (32.4)	72 (32.0)	17 (36.2)	
**ECOG**				**0.047c**
0	166 (60.4)	139 (61.0)	27 (57.5)	
1	84 (30.6)	73 (32.0)	11 (23.4)	
≥2	12 (4.4)	7 (3.1)	5 (10.6)	
Unknown	13 (4.7)	9 (4.0)	4 (8.51)	

Overview of the clinical characteristics of all included patients with advanced ovarian cancer (n = 275). A total of 47 elderly patients ≥70 years are opposed to 228 patients younger than 70 years.

a Man-Whitney-U-test, b Chi^2^-test, c Fisher’s exact test.

* other = clear cell, endometroid, mixed cell, undifferentiated histology.

*FIGO* International Federation of Gynecology and Obstetrics.

*ECOG* Eastern Cooperative Oncology Group performance status.

Despite the non-randomized setting both patient cohorts (<70 years vs. ≥70 years) were well balanced in terms of tumor stage, grading, histological subtype and presence of ascites or peritoneal carcinomatosis (Table 1). The majority of patients (71.3%) were diagnosed in FIGO stage IIIC and 17.5% in FIGO stage IV. Classification of stage IV was mainly because of malignant pleural effusion (35%) or metastases to the liver parenchyma (25%). In the elderly subgroup FIGO stages were slightly higher (FIGO IIIC 66.0%, FIGO IV in 23.4%) compared to younger patients, however, this difference was not statistically significant, p = 0.801. Contrarily, nodal status differed significantly between the age groups with a higher rate of unknown lymph-node status (pNx) in elderly patients (44.7% vs. 20.2%, p < 0.001). Their performance status expressed by the ECOG (Eastern Cooperative Oncology Group) score was significantly poorer compared to younger patients (ECOG ≥2 in 10.6% vs. 3.1%, p = 0.047).

In addition, analysis of treatment-related patient characteristics (Table 2) revealed that intraoperative surgical procedures were comparable except for distinct differences regarding the frequency of adnectomy (83.0% vs. 96.4%, p = 0.038), hysterectomy (76.6% vs. 93.0% p = 0.006) and pelvic (42.6% vs. 75%, p < 0.001) as well as para-aortic (38.3% vs. 72.8%, p < 0.001) lymphadenectomy in elderly compared to younger patients, reflected by different nodal status as previously described. There were no significant differences in terms of upper abdominal surgery such as bowel resection or splenectomy but elderly patients underwent interval debulking after neoadjuvant chemotherapy significantly more frequent (p = 0.019). Of note, rates of postoperative residual tumor differed singnificantly (p = 0.029) with higher rates of suboptimal surgical outcome in elderly patients (44.7% vs. 28.5%). Intraoperative complication rates (bladder, liver capsula or spleen lesion, cardiac ischemia, large vessel laceration, mass transfusions) were comparable between both groups (p = 0.532) and the proportion of patients with an unremarkable postoperative clinical course did also not differ between the two age groups (p = 0.495). In both groups none of the patients died within 30 postoperative days (p = 1.00) while the 60-day mortality rate was slightly but significantly higher in elderly patients with 2.13% compared to 0.44% in patients <70 years (p < 0.001, Table 2).

**Table 2:** Treatment-related patient characteristics

	**Total (%) n = 275**	**Age <70 years (%) n = 228**	**Age ≥ 70 years (%) n = 47**	**p value for group differences**
**Type of surgery**				**0.019b**
Primary cytoreduction	226 (82.2)	193 (84.6)	30 (70.2)	
Interval debulking	49 (17.8)	35 (15.4)	14 (29.8)	
**Surgical interventions**				
Adnectomy	259 (94.2)	220 (96.4)	39 (83.0)	**0.038b**
Hysterectomy	248 (90.2)	212 (93.0)	36 (76.6)	**0.006b**
Omentectomy	255 (92.7)	212 (93.0)	43 (91.5)	0.720b
Pelvic LAE	191 (69.5)	171 (75.0)	20 (42.6)	**<0.001b**
Paraaortic LAE	184 (66.9)	166 (72.8)	18 (38.3)	**<0.001b**
Appendectomy	110 (40.0)	94 (41.2)	16 (34.0)	0.327b
Splenectomy	22 (8.0)	19 (8.3)	3 (6.4)	1.000b
Large bowel resection	104 (37.8)	87 (38.2)	17 (36.2)	0.798b
Small bowel resection	37 (13.4)	27 (11.8)	10 (21.3)	0.084b
**Postop. residual tumor**				**0.029b**
Microscopic	189 (68.7)	163 (71.5)	26 (55.3)	
Macroscopic	86 (31.3)	65 (28.5)	21 (44.7)	
**Intraoperative complications**				0.532c
Yes	20 (7.2)	16 (7.0)	4 (8.5)	
No	247 (89.9)	209 (91.7)	38 (80.9)	
Unknown	8 (2.91)	3 (1.3)	5 (10.6)	
**Postoperative complications**				0.495b
Yes	95 (34.6)	82 (36.0)	13 (27.7)	
No	172 (62.6)	143 (62.7)	29 (61.7)	
Unknown	8 (2.9)	3 (1.3)	5 (10.6)	
**Mortality rates**				
30d-mortality rate	0.0 %	0.0 %	0.0 %	1.000d
60d-mortality rate	1.1 %	0.4 %	2.1 %	**<0.001d**
**Chemotherapy**				
courses (median, range)	6 (1–8)	6 (1–8)	6 (2–6)	0.156a
Time to chemo after surgery (median, range)	34 (5–188)	34 (5–188)	31.5 (9–127)	0.368a
Treatment concept				**0.019b**
- Neoadjuvant	49 (17.8)	35 (15.4)	14 (29.8)	
- Adjuvant	226 (82.2)	193 (84.6)	30 (70.2)	
Treatment regimen				**<0.001c**
- Platinum-based combination	260 (94.5)	223 (97.8)	37 (78.7)	
- Platinum monotherapy	15 (5.5)	5 (2.2)	10 (21.3)	
**Response to chemotherapy**	205 (74.5)	178 (78.4)	27 (57.4)	**0.003b**
**Optimal oncologic treatment** (no residual tumor + combination therapy)				**<0.001b**
Yes	179 (65.1)	160 (70.1)	19 (40.4)	
No	96 (34.1)	68 (29.8)	28 (59.6)	
**Survival***(months)*				
Median PFS	19	20	12	**0.022d**
Median OS	48	64	30	**<0.001d**

Detailed overview of surgical procedures, chemotherapy and prognostic aspects. Parameters were separately analyzed for elderly patients ≥70 years and patients younger than 70 years.

a Man-Whitney-U-test, b Chi^2^-test, c Fisher’s exact test, d log-rank test.

LAE = lymphadenectomy, PFS = progression free survival, OS = overall survival.

All included patients received platinum-based chemotherapy according to the inclusion criteria but there were significantly more elderly patients receiving single-agent platinum instead of a combination regimen (21.3% vs. 2.2% of the younger patients, p < 0.001). Approximately 3/4 of the younger patients experienced clinical response to primary chemotherapy compared a significantly lower rate in the elderly cohort (57.4% vs. 78.5%, p = 0.003). Taken together only 40.4% of the elderly compared to 70.1% of the younger patients received optimal oncologic treatment as it is considered for the overall patient population consisting of complete tumor resection and platinum-based combination chemotherapy (p < 0.001, Table 2).

**Figure 1:**
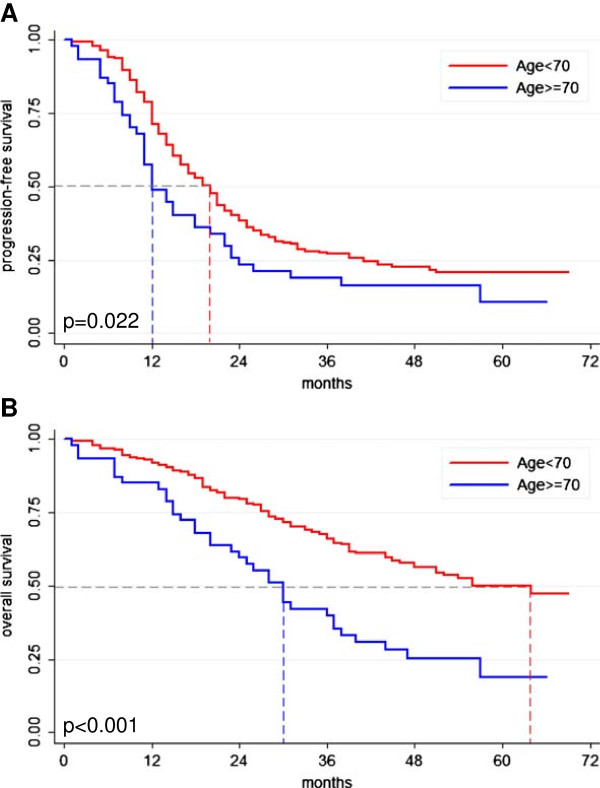
**Prognosis of ovarian cancer patients according to age.** Kaplan-Meier curves demonstrating significantly impaired prognosis for elderly ovarian cancer patients regarding progression-free survival (PFS) (**A**; 12 vs. 20 months, p = 0.022) and overall survival (OS) (**B**; 30 vs. 64 months, p < 0.001). Statistical comparison by log rank test.

**Figure 2:**
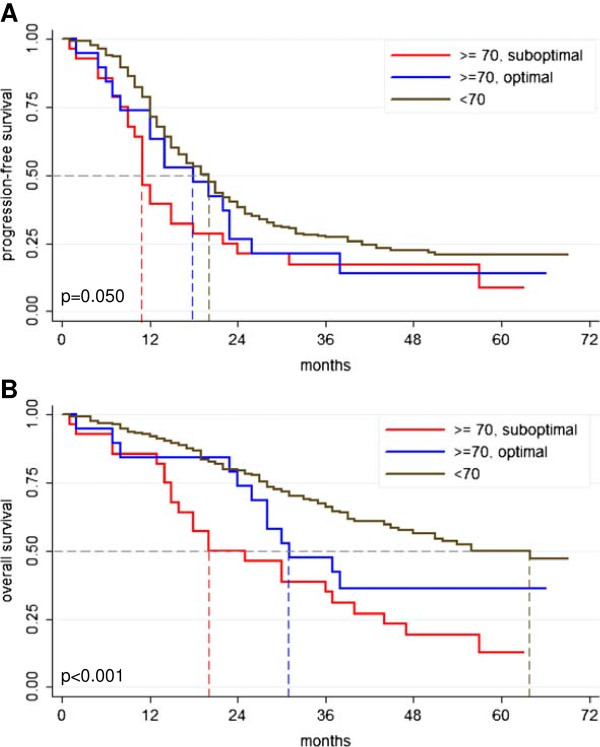
**Prognosis of ovarian cancer patients according to age.** Kaplan-Meier curves regarding progression-free (PFS) and overall survival (OS) separately analyzed for patients ≥70 years with suboptimal or optimal oncologic treatment opposed to patients younger than 70 years (**A**; PFS: 11 months vs. 18 months vs. 20 months, p = 0.05; **B**; OS: 20 months vs. 31 months vs. 64 months, p < 0.001). Statistical comparison by log rank test. *optimal = complete tumor resection and platinum-based chemotherapy.*

Outcome of the age subgroups differed significantly with median progression-free survival (PFS) of 12 vs. 20 months for elderly patients compared to younger patients (p = 0.022, Figure 1A, Table 2) and 30 vs. 64 months in terms of median overall survival (OS) (p < 0.001, Figure 1B, Table 2). Elderly patients with optimal oncologic management had an improved prognosis regarding PFS and OS compared to elderly patients receiving sub-optimal oncologic treatment (median PFS 18 vs. 11 months, p = 0.05; median OS 31 vs. 20 months, p < 0.001; Figure 2). Of note, the clinical course of the optimally treated elderly subgroup approached to that of the cohort of younger patients for PFS when Kaplan-Meier method was applied (Figure 2A).

Despite the highly significant differences in prognosis in univariate analysis, age itself was not an independent prognostic factor for PFS in multivariate analysis (Table 3). However, regarding OS age remained highly significant in the multivariate analysis in the presence of other prognostic factors (Table 3). Concentrating on the patient group aged 70 and older FIGO stage and ECOG performance status were independent prognostic factors regarding PFS (ECOG ≥1 vs. 0 HR 2.64, p = 0.048; Table 4). Receiving mono-chemotherapy instead of combination chemotherapy was almost significantly related to worse outcome regarding OS (HR 2.80, p = 0.055; Table 4).

**Table 3:** Multivariate analysis of prognostic factors for the overall patient cohort

	**Progression-free survival**				**Overall survival**			
	**HR**	**95% CI**		**p value**	**HR**	**95% CI**		**p value**
**FIGO stage** IV vs. II-IIIC	2.169	1.489	3.159	**<0.001**	1.730	1.135	2.635	**0.011**
**Grading**								
G2 vs. G1	1.809	0.711	4.604	0.213	1.681	0.560	5.049	0.355
G3 vs. G1	1.835	0.742	4.535	0.189	1.823	0.638	5.214	0.262
**Lymph-node status**								
N1 vs. N0	1.046	0.732	1.494	0.805	1.495	0.913	2.448	0.110
Nx vs. N0	1.455	0.949	2.231	0.085	2.018	1.125	3.619	**0.018**
**Residual tumor** (yes vs. no)	1.384	0.995	1.926	0.054	1.568	1.038	2.369	**0.033**
**Peritoneal carcinomatosis** (yes vs. no)	2.600	1.811	3.733	**<0.001**	2.528	1.538	4.153	**<0.001**
**Histologic subtype**								
Serous vs. mucinous	0.852	0.170	2.915	0.630	0.386	0.090	1.658	0.201
Serous vs. other	0.833	0.531	1.305	0.766	0.735	0.432	1.253	0.258
**Age** (<70y vs. ≥70y)	1.226	0.853	1.761	0.272	1.836	1.207	2.794	**0.005**
**ECOG**								
≥1 vs. 0	1.137	0.824	1.571	0.434	1.315	0.842	2.052	0.229
Unknown vs. 0	0.852	0.409	1.775	0.669	1.185	0.457	3.070	0.727

Detailed analysis of possible prognostic factors regarding their statistical significance and independence by cox regression model for the overall patient cohort (n = 275).

**Table 4:** Multivariate analysis of prognostic factors for elderly patients (≥70 years)

	**Progression-free survival**				**Overall survival**			
	**HR**	**95% CI**		**p value**	**HR**	**95% CI**		**p value**
**FIGO stage** IV vs. II-IIIC	2.790	1.007	6.905	**0.048**	2.083	0.792	5.480	0.137
**Grading**								
G2 vs. G1	0.351	0.020	6.312	0.478	2.440	0.139	42.786	0.542
G3 vs. G1	2.023	0.144	28.437	0.601	5.165	0.368	72.453	0.223
**Lymph-node status**								
N1 vs. N0	0.277	0.050	1.532	0.141	0.191	0.260	1.404	0.104
Nx vs. N0	0.177	0.023	1.362	0.096	0.176	0.018	1.744	0.138
**Residual tumor** (yes vs. no)	0.635	0.233	1.734	0.376	0.919	0.321	2.634	0.876
**Peritoneal carcinomatosis** (yes vs. no)	1.752	0.734	4.183	0.207	0.563	0.612	3.317	0.490
**Histologic subtype**								
Serous vs. other	0.120	0.008	1.855	0.129	0.073	0.005	0.995	0.050
**Chemotherapy**								
Mono vs. combination	0.924	0.334	2.552	0.878	2.800	0.978	8.012	0.055
**ECOG**								
≥1 vs. 0	2.637	1.007	6.905	**0.048**	1.819	0.651	5.081	0.254
Unknown vs. 0	2.682	0.435	16.670	0.287	2.697	0.405	17.943	0.305

Detailed analysis of possible prognostic factors regarding their statistical significance and independence by cox regression model for the cohort of patients age 70 and older (n = 47).

## Discussion

In this European multicenter study aiming to identify and verify clinical and molecular prognostic factors for ovarian cancer, patients with advanced disease were prospectively included resulting in a well characterized cohort of patients treated in high-volume gynecologic oncology centers. In our study prognosis of patients ≥70 was significantly impaired. This report demonstrates that patients aged ≥ 70 years do frequently not receive optimal multimodal therapy despite treatment in specialized cancer centers, possibly contributing to the observed impaired outcome.

Debulking surgery with the intent of complete tumor resection is frequently feasible, even in advanced disease stages [4]. However, cytoreduction of advanced disease often requires radical surgical steps like bowel resection, upper abdominal surgery or pelvic as well as para-aortic lymphadenectomy. In this context, a major concern of surgeons towards elderly patients is the fear of a higher complication and mortality rate as revealed by a retrospective analysis from the Washington State Hospital in 2009 with rising complication rates for abdominal surgery according to age (65–69 years, 14.6%; 70–74 years, 16.1%; 75–79 years, 18.8%; 80–84 years, 19.9%; 85–89 years, 22.6%; p < 0.001) [14]. Validated selection criteria regarding reasonable treatment decisions for elderly patients are currently not available. In a systematic review, however, focusing on primary cytoreductive surgery for advanced ovarian cancer, a 30-day-mortalitity rate between 0 and 5.9% with a median of 2.7% has been reported by Gerestein et al. [15]. Although age-specific information in the investigated studies were sparse, postoperative mortality seemed to be slightly elevated in elderly patients (5.4-11.7%) albeit still acceptable [15]. This was recently confirmed by Thrall et al. with a 30-day mortality rate of 5.6% for elective surgeries in elderly ovarian cancer patients [16]. In a retrospective investigation from Maryland, mortality rates of elderly patients were reported to be lower in high-volume departments compared to departments with less than 20 cytoreductive surgeries for ovarian cancer per year [17]. In the present analysis no deaths were registered within the first 30 postoperative days while the 60-day mortality rate of 2.1% was only slightly elevated for elderly patients compared to the younger patient group (0.4%). In addition, perioperative complication rates in this analysis were comparable with previous reports [18,19] and did not show significant differences between younger and elderly patients despite significantly poorer performance status in the elderly group. These results underline that in high volume departments with multidisciplinary treatment and distinct experience for ovarian cancer, higher age itself as well as complication-concerns should not be a reason to withhold optimal surgical treatment from the patients.

Although intraoperative procedures were equally distributed between elderly and younger patients except for adnectomy, hysterectomy as well as pelvic and para-aortic lymphadenectomy, complete macroscopic tumor resection was less frequently achieved in elderly patients. In addition, elderly patients were more likely to receive single-agent chemotherapy which was almost not applied to younger patients and showed a prognostic trend for worse outcome regarding OS in elderly patients. Consecutively, the rate of sub-optimal oncologic management was significantly higher in elderly patients although a general feasibility of multimodal treatment has been previously proven for these patients [20]. As rates for complete macroscopic tumor resection exceed the frequency of pelvic or para-aortic lymphadenectomy in the elderly cohort, in some of these cases lymphadenectomy was not performed to spare the elderly the morbidity of the procedures themselves. So far, rationales to hesitate applying radical surgical and systemic therapy to elderly patients are a higher prevalence of co-morbidities and a general lowered life expectancy [21]. This is aggravated by the low level of evidence on specific treatment strategies for elderly patients [21,22]. A meta-analysis of randomized controlled studies by Hilpert et al. concluded that the investigators’ intention to maintain treatment of elderly patients within the study protocol despite protocol-predefined options as dose reductions, cycle delay or supportive therapy is limited [3]. Furthermore, elderly patients in our cohort more frequently underwent interval debulking surgery after neodadjuvant chemotherapy shown to be related with worse outcome compared to primary debulking surgery in this non- randomized OVCAD cohort [23].

All these observations might explain the fact that age itself could not convincingly be confirmed as an independent prognostic factor in the present analysis. Although several previously published studies attributed age to be independently related with unfavourable outcome in elderly patients [5-7], a large retrospective analysis from Denmark described that the prognostic significance diminished for patients receiving optimal oncologic treatment [8]. This is in line with the present analysis, in which only patients being treated in high-volume departments with high standards of quality and a long year experience were included. For the elderly cohort, ECOG performance status was prognostically significant with a favorable prognosis for patients without significant comorbidities compared to patients with impaired performance status (ECOG ≥1). This further underlines that chronical age itself is not an ideal factor to stratify patients. As demonstrated, health and performance status of the patients (indicating their ‘biological age’) should rather be considered by gynecologic oncologists before treatment decisions are made. Although ECOG performance status as well as ASA (American Society for Anesthesiologists) scale reflecting co-morbidities might roughly estimate the functional status of our patients, further, more thorough assessment strategies are highly warranted. Within the international PACE study (Preoperative Assessment of Cancer in the Elderly) a geriatric assessment tool for elderly cancer patients (>70 years) was prospectively evaluated and recently published [24]. In this trial, different validated evaluation tests as the Comprehensive Geriatric Assessment (CGA), the Brief Fatigue Inventory and the ASA scale were combined to conceive a reliable picture of the health, functional and cognitive status of elderly patients [25]. This approach was able to predict the risk of both postsurgical complications and extended hospital stay and appears to be a helpful additional tool for decision making [26]. However, predictive accuracy of this approach needs to be further validated in future studies.

Regarding the obvious underrepresentation of elderly patients in current clinical trials, studies specifically concentrating on the distinct needs and expectations of elderly patients are highly desirable [3,27]. These trials should also consider that apart from effects on overall survival, improved PFS would be the more important therapeutic goal for elderly patients. As older patients are more likely to die from other causes than cancer, potential effects on overall survival might diminish [13]. Moreover, an improved PFS may lead to a better quality of the remaining life which would otherwise be significantly impaired by symptomatic advanced ovarian cancer. In general, specific trials for elderly and frail patients might serve as ideal context to establish new therapeutic endpoints like patient reported outcomes (PRO) [28].

The Gynecologic Cancer Intergroup (GCIG) is currently planning a prospective trial specifically tailored for elderly women with ovarian cancer (EWOC) that evaluates optimal treatment strategies for elderly patients with the disease but will also address possible new endpoints.

## Conclusions

Elderly patients with ovarian cancer are often treated less radically than younger patients which has been confirmed in this European multicenter study. Their outcome is significantly impaired despite no consistent prognostic effect of age.

Notwithstanding this treatment reality, age itself should not be a reason to withhold optimal oncologic treatment from elderly patients. Comprehensive preoperative assessment should be performed considering the biological age and functional status of the patients. With this information individualized treatment plans can be defined accounting for co-morbidities and expected complications.

## Competing interests

The authors declare they have no competing interests.

## Authors’ contributions

FT, LW and SM were responsible for the concept and design of the presented study, analyzed the data and prepared the manuscript. CE participated in the study design and performed the statistical analysis. All authors were involved in the data acquisition and quality control for the trial. All authors supported the manuscript editing, read and approved the final manuscript.
